# Special issue 100th anniversary *Cell and Tissue Research*

**DOI:** 10.1007/s00441-025-03972-4

**Published:** 2025-05-01

**Authors:** Horst-Werner Korf, David P. Kelsell, Vera Kozjak-Pavlovic

**Affiliations:** 1 Institute for Anatomy 1, Heinrich Heine University Düsseldorf, Düsseldorf, Germany; 2 Faculty of Medicine and DentistryBlizard Institute, Queen Mary University of London, London, UK; 3 Department of Microbiology, Julius Maximilian University of Würzburg, Würzburg, Germany

2024 has marked *Cell* *and Tissue Research*’s 100th year of publication which we are delighted to celebrate with this special anniversary issue.

The journal was founded under the name of *Zeitschrift für Zellforschung und mikroskopische Anatomie* in 1924 (Fig. 1; Unsicker 2025) as a multilingual journal which published papers in German, French, and English. In 1974, the journal was transformed into *Cell and Tissue Research* (CTR), publishing papers in English only to enhance international visibility of the journal. Within the last century, CTR is the longest-running active journal dedicated to cell biology serving as a prominent platform to communicate timely and novel results in cell biology and microscopic anatomy across species. From the very beginning, the journal has consistently published cutting-edge research and has also provided major technical advances in the fields of microscopy and tissue culture.

**Fig. 1:**
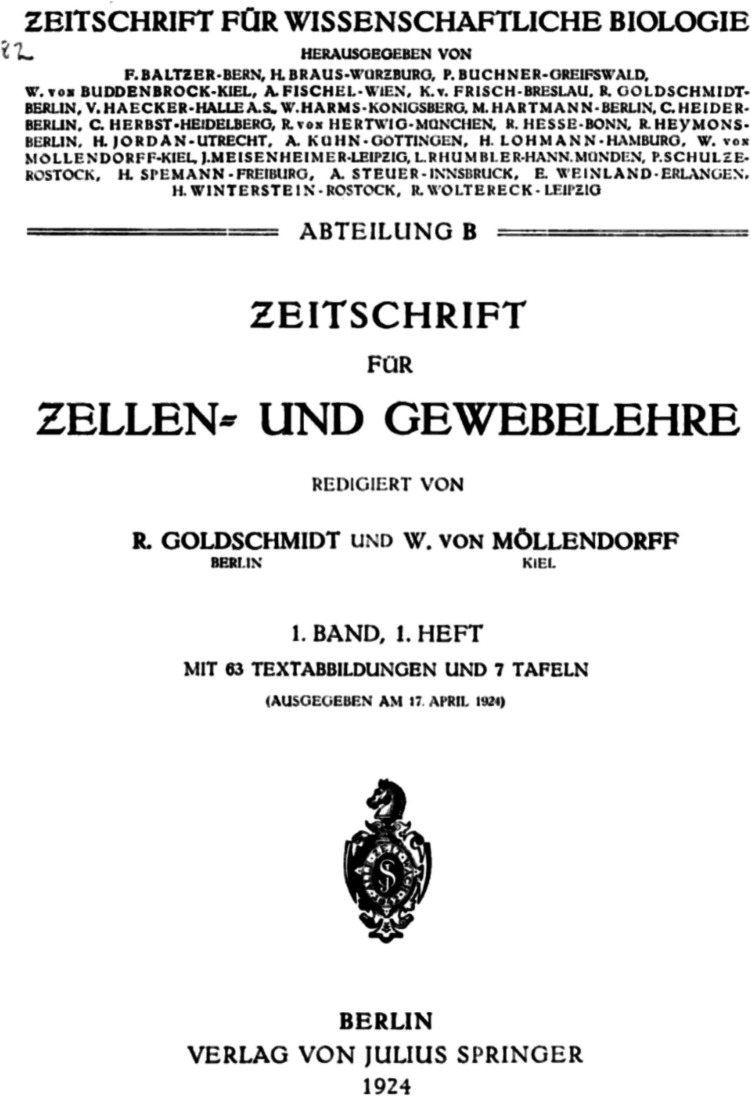
Front page of the first issue of the Journal

In this special issue, examples of such discoveries are described from the sections of Reproduction, Immunology, and Neuroendocrinology.

## Reproductive biology

As outlined by Meinhardt and Sutovsky (2024), CTR has published significant contributions in the fields of spermatology and embryology focusing on both plant and animal sperm cells. This review revisits 100 years of research on the male germ cells and fertility in humans and animals and offers a perspective on the current state and future directions of the andrology field. Early technological advances in light and electron microscopy enabled descriptive studies that ushered in the era of mechanistic, biochemistry-based inquiry focused on the understanding of physiological sperm processes such as sperm capacitation, acrosomal exocytosis, and sperm-egg interactions. In the last 20 years, progress in flow cytometry, cell imaging, and `omics´ revealed new information on sperm proteome, transcriptome, metabolome, and overall phenome of fertile and infertile spermatozoa. Going back to the journal’s roots, recent advances in male germ cell isolation, transplantation, modification, and cryopreservation have been discussed on the pages of CTR. Newest trends such as gene editing and artificial intelligence/machine learning are now making inroads into andrological inquiry and assisted reproductive therapy of male infertility.

## Immunology/Inflammation

Graham et al. (2024) highlight the importance of the Kupffer cell and explore the history of the Kupffer cell in the context of infection beginning with its discovery to the present day. Karl Wilhelm von Kupffer discovered the cells in 1876 and denominated them as “Sternzellen.” Since their discovery as the primary macrophages of the liver, an in-depth understanding of the identity, functions, and influential role of Kupffer cells, particularly in infection, has been obtained. Kupffer cells perform important tissue-specific functions in homeostasis and disease. Stationary in the sinusoids of the liver, Kupffer cells have a high phagocytic capacity and are adept in clearing the bloodstream of foreign material, toxins, and pathogens. Thus, they are indispensable to host defence and prevent the dissemination of bacteria during infections.

## Neuroendocrinology

**Fig. 2:**
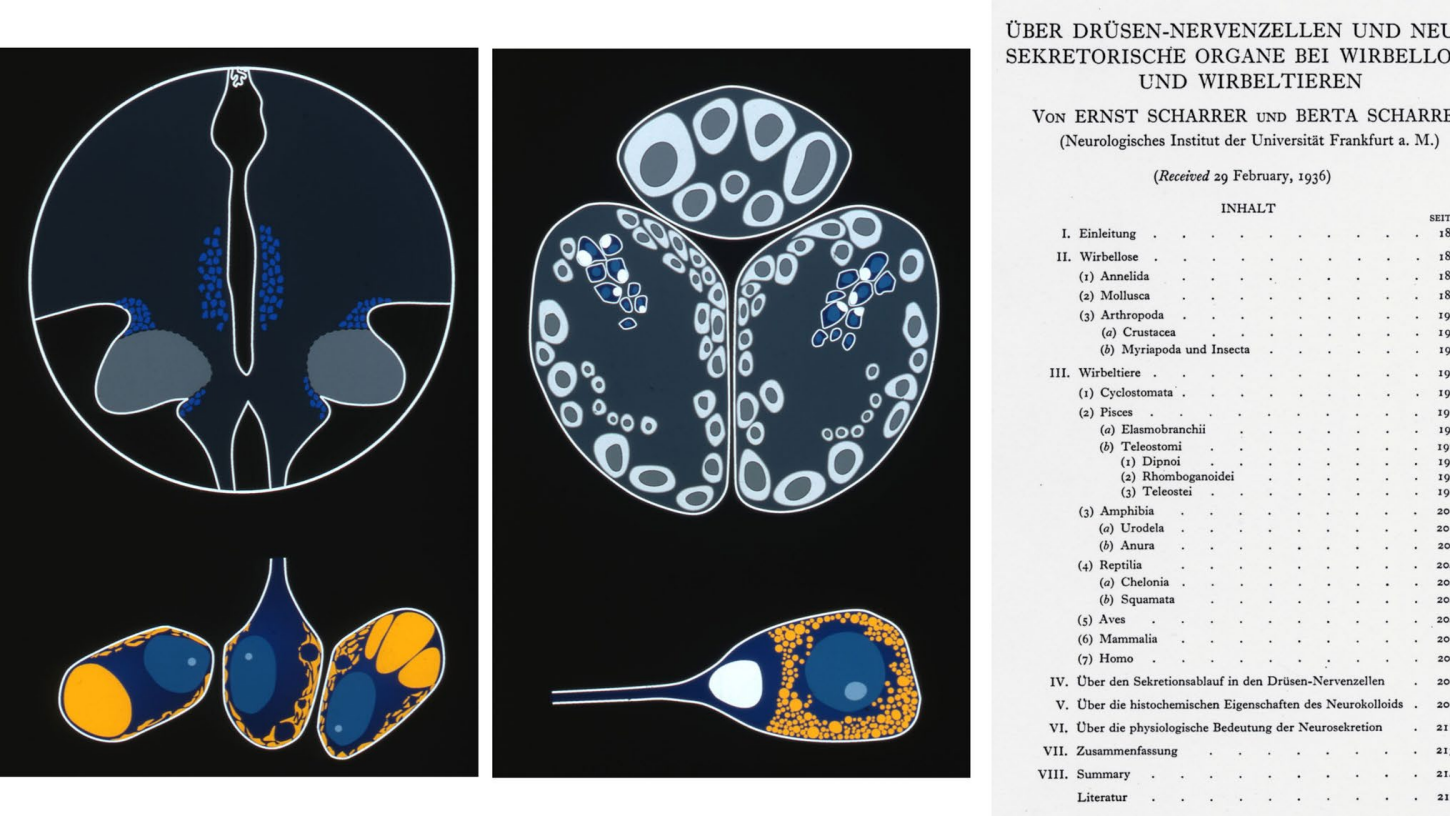
Diagrammatic representation of secretory neurons in vertebrates (left) and invertebrates (middle). The microscopic appearance of the cells is shown at the bottom. Front page of the paper by Ernst and Berta Scharrer (1937) presenting the first, nearly prophetic concept on neurosecretion and neuroendocrinology

**Fig. 3:**
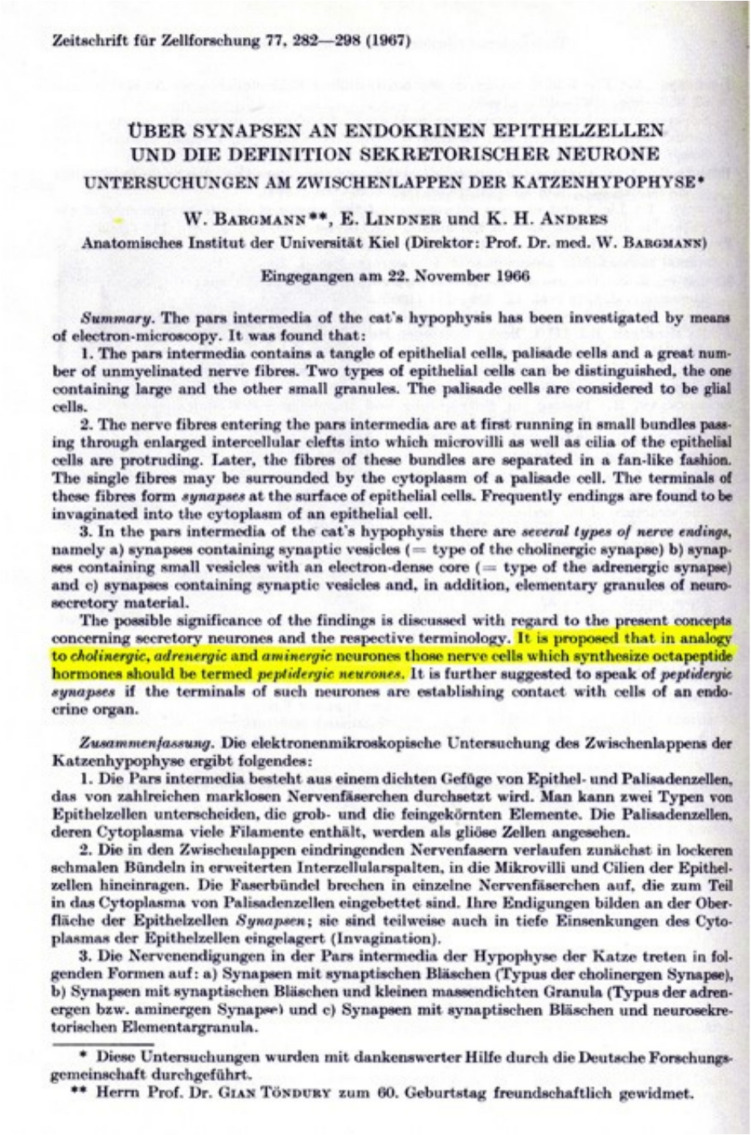
Front page of the paper by Bargmann et al. (1967), coining the term “peptidergic neuron”

Rodriguez et al. (2025) present early roots in neuroendocrinology. The development of the concept of neuroendocrinology has greatly benefitted from the comparative approach investigating vertebrates and invertebrates (Fig. 2) (Scharrer 1952, 1963; Scharrer and Scharrer 1937, 1954, 1963; Oksche et al. 1959). Milestones published in the journal were the visualization of the magnocellular hypothalamic system and the discovery of the hypothalamo-hypophysial tract by means of the Gomori technique (Bargmann 1949), coining the term peptidergic neuron (Fig. 3) (Bargmann et al. 1967), and the first immunocytochemical investigations of the hypothalamo-hypophysial system using antibodies against neurophysin, vasopressin, and oxytocin (Vandesande and Dierickx 1975, Vandesande et al. 1974).

In an associated publication, Nässel (2025) reviews 50 years of research on insect neuropeptide and peptide hormone (collectively abbreviated NPH) signaling, initiated by the sequencing of proctolin in 1975. Research before the sequencing of the *Drosophila* genome aimed at identification of novel NPHs by biochemical means and mapping their distribution in neurons, neurosecretory cells, and endocrine cells of the intestine. Functional studies of NPHs dealt with hormonal aspects of peptides and many employed ex vivo assays. A new era followed after the annotation of the *Drosophila* genome, and more specifically of the NPHs and their receptors in *Drosophila* and other insects. NPH ligands were attributed to orphan receptors and NPHs were localized by means of improved detection methods. Important advances were made with introduction of a rich repertoire of innovative molecular genetic approaches to localize and interfere with expression or function of NPHs and their receptors. These methods enabled cell- or circuit-specific interference with NPH signaling for in vivo assays to determine roles in behavior and physiology, imaging of neuronal activity, and analysis of connectivity in peptidergic circuits. NPHs were found to play multiple roles in development, physiology, and behavior. Importantly, we can now appreciate the pleiotropic functions of NPHs, as well as the functional peptidergic “networks” where state-dependent NPH signaling ensures behavioral plasticity and systemic homeostasis. Future studies can now model state/context-dependent neuronal signaling in networks of the brain taking into account both synaptic signaling and NPH-mediated neuromodulation.

## Non-visual photoreceptors, photoneuroendocrinology, and circadian biology

The journal has published pioneering morphofunctional studies on extraretinal photoreceptors (Oksche and Kirchstein 1967, 1968) which represent non-visual photoreceptors serving to orientate in time, rather than in space. These photoreceptors represent the input pathway of the photoneuroendocrine system as defined by Ernst Scharrer (1964). Notably, the pineal specific cells have undergone a transformation from true pineal photoreceptors in anamniotes to neuroendocrine pinealocytes in mammals. Also the innervation of the pineal organ has changed: while pinealofugal nerve fibers (pineal tract) leave the directly photoreceptive pineal organs of anamniotes, the pinealopetal sympathetic innervation increases during phylogenetic development and becomes the most important input pathway to the neuroendocrine pineal organ of mammals (Kappers 1960). After the discovery of clock genes and the molecular clock work in *Drosophila* and mammals, photoneuroendocrinology has evolved into circadian biology, which is of fundamental importance for health and disease (cf. Korf 2024, this issue).

## Special issues/Topical collections

Special issues were introduced by our past Coordinating Editor, Prof. Dr. Klaus Unsicker, who succeeded Andreas Oksche in 1996 and served this role until 2023. Special Issues have summarized cutting-edge research during the last decades (Table 1).

**Table 1:** Cell and Tissue Research: Special Issues list

Title, volume	Guest editors	Editor	MS	Pages
Glial cell line-derived neurotrophic factor (Vol. 286, No. 2, 1996)	K. Unsicker	Unsicker	12	104
Molecular bases of axonal growth and pathfindings (Vol. 290, No. 2, 1997)	U. Drescher, A. Faissner, R. Klein, FG. Rathjen, C. Stürmer	Unsicker	34	285
Molecular bases of limb and muscle development (Vol. 296, No. 1, 1999)	R. Zeller	Unsicker	22	219
Apoptosis 2000 (Vol. 301, No. 1, 2000)	J. Reed, M. Weller	Unsicker	15	204
Recent advances in developmental neuroscience (Vol. 305, No. 2, 2001)	K. Unsicker	Unsicker	12	115
The circadian system: circuits – cells – clock genes (Vol. 309, No. 1, 2002)	H.-W. Korf J.H. Stehle	Korf	18	199
Vasculogenesis and angiogenesis (Vol. 314, No. 1, 2003)	R. Adams	Unsicker	18	177
The dopaminergic nigrostriatal system: development, physiology, disease (Vol. 318, No. 1, 2004)	O. von Bohlen und Halbach, K. Krieglstein, A. Schober, JB. Schulz	Unsicker	26	288
Reproduction, development, and the early origins of adult disease (Vol. 322, No. 1, 2005)	AE. Drummond, M. Wlodek	Risbridger	21	181
The synapse – Recent advances (Vol. 326, No. 2, 2006)	M. Frotscher, E. Gundelfinger P. Jonas, E. Neher, P. Seeburg	Unsicker	34	468
Stem cells: established facts, open issues, and future directions (Vol. 331, No. 1, 2008)	G. Kuhn, O. Brüstle, U. Martens, A. Wobus	Unsicker	28	372
Endothelial cell biology and pathology (Vol. 335, No. 1, 2009)	E. Dejana, H. Wolburg, M. Simionescu	Franke	20	300
Cell interactions with the extracellular matrix (Vol. 339, No. 1, 2010)	L. Bruckner-Tuderman, K. Von Der Mark	Pihlajaniemi	21	280
Innate immunity (Vol. 343, No.1, 2011)	B. Singh, G. Mutwiri, P. Griebel	Singh	21	261
TGF-ß in aging and disease (Vol. 347, No. 1, January 2012)	K. Krieglstein, K. Miyazono, P. ten Dijke	Unsicker	27	301
Endogenous musculoskeletal tissue regeneration (Vol. 347. No. 3, March 2012)	D.W. Hutmacher, G. Duda, R.E. Guldberg	Unsicker	27	345
Molecular Biology meets Cardiology (Special Workshop “Heidelberg Heart II”) (Vol. 348, No. 2, May 2012)	W.W. Franke, W. Birchmeier	Franke	10	121
Molecular Bases of Neural Repair Mechanisms (Vol. 349, No.1, July 2012)	H.W. Müller, M. Sendtner, M. Bähr	Unsicker	30	404
Cell biology solves mysteries of reproduction (Vol. 349, No. 3, September 2012)	P. Sutovsky	Sutovsky	19	264
Current insights into protease dynamics in human epithelial disease and barrier function (Vol. 351, No. 2, February 2013)	M.A. Curtis, D.P. Kelsell	Kelsell	12	139
Cell-to-cell communication: current views and future perspectives (Vol. 352, No. 1, April 2013)	H–H. Gerdes, R. Pepperkok	Unsicker	13	177
Neuroprotection in Glaucoma (Vol. 353, No. 2, August 2013)	E.R. Tamm, F. Grehn, N. Pfeiffer	Unsicker	15	153
Rodent models of psychiatric disorders—practical considerations (Vol. 354, No. 1, October 2013)	P. Gass, C. Wotjak	Unsicker	24	330
Between sealing and leakiness: molecular dynamics of the endothelium to maintain and regulate barrier function (Vol. 355, No. 3, March 2014)	H. Schnittler	Unsicker	20	256
Epigenetics: Development, Dynamics and Disease (Vol. 356, No. 3, August 2014)	T. Vogel, S. Lassmann	Unsicker	18	213
Dysfunction of neuronal calcium signaling in aging and disease (Vol. 357, No. 2, August 2014)	A.M.M. Oliveira, H.Bading, D. Mauceri	Unsicker	10	122
Deciphering the core instructions of neuronal differentiation (Vol. 359, No. 1, January 2015)	U. Ernsberger	Unsicker	25	384
Quantitative Techniques for Imaging Cells and Tissues (Vol. 360, No. 1, April 2015)	C. von Bartheld, F. Wouters	Unsicker	14	194
Junctions in human health and inherited disease (Vol. 360, No. 3, June 2015)	S. Getsios, D. P. Kelsell, A. Forge	Kelsell	25	348
Auditory system: development, genetics, function, aging, and diseases (Vol. 361, No. 1, July 2015)	B. Fritzsch, M. Knipper, E. Friauf	Unsicker	25	399
Reproductive systems biology tackles global issues (Vol. 363, No. 1, January 2016)	P. Sutovsky, A.S. Cupp, W. Thompson, M. Baker	Unsicker	24	312
Wound healing and fibrosis – two sides of the same coin (Vol. 365, No. 3, September 2016)	D. Gullberg, D. Kletsas, T. Pihlajaniemi	Pihlajaniemi	19	241
Recent Advances in Mitochondrial Biology—Integrated Aspects (Vol. 367, No. 1, January 2017)	C. Meisinger, C. Hunte	Unsicker​	13	159
Development, remodeling and regeneration of the lung (Vol. 367, No. 3, March 2017)	C. Muehlfeld, M. Ochs, B. Singh	Singh	25	362
Genetic Kidney Diseases (Vol. 369, No 1, July 2017)	T. Huber, H. Holthofer	Unsicker	21	244
Neural stem cells: developmental mechanisms and disease modeling (Vol. 371, No 1, January 2018)	X. Zhao, D. Moore	Zhao	17	212
Neutrophil Biology (Vol. 371, No 3, March 2018)	S. Liao, C. Jenne, B. Singh	Singh	23	253
The sympathetic nervous system: malignancy, disease, and novel functions (Vol. 372, No 2, May 2018)	K. Huber, I. Janoueix-Lerosey, W. Kummer, H. Rohrer, A.S. Tischler	Unsicker	23	280
Parkinson’s disease: Molecules, cells, and circuitries (Vol. 373, No 1, July 2018)	H. Braak, K. Del Tredici-Braak, T. Gasser	Unsicker	24	336
Recent advances in hippocampal structure and function (Vol. 373, No 3, September 2018)	O von Bohlen und Halbach, A. Draguhn, J. Storm-Mathisen	Unsicker	13	220
Towards new frontiers in neuroendocrinology: A tribute to Peter H. Seeburg (Vol. 375, No 1, January 2019)	V. Grinevich, Heidelberg-Mannheim and G. F. Jirikowski, Jena	Unsicker	27	327
Depression and antidepressant action—from molecules to neworks (Vol. 377, No 1, July 2019)	Tomi Rantamäki, Ipek Yalcin	Unsicker	9	124
Structure, Development and Evolution of the Digestive System (Vol. 377, No 3, September 2019)	Volker Hartenstein, Pedro Martinez-Serra, Barcelona	Hartenstein	15	258
“Tribute to Werner W. Franke” (Vol. 379, No 1, January 2020)	K. Unsicker	Unsicker	19	222
Animal Models (Vol. 380, No 2, May 2020)	D. Meyerholz, A.P. Beck, B. Singh	Singh	12	209
Special Issue on Cell Biology of Neurotrophic Factors (Vol. 382, No 1, October 2020)	Mart Saarma, William Mobley, Volkmar Leßmann	Unsicker	15 + 1	200
Special Issue “Olfactory Coding and Circuitries” (Vol. 383, No 1, January 2021)	Silke Sachse and Ivan Manzini	Unsicker	40 + 1	595
Immune-Mediated Kidney Diseases (Vol. 385, No 2, August 2021)	Ulf Panzer and Tobias B. Huber	Unsicker	16 + 1	223

## Human pluripotent stem cell technologies and translational neuroscience

A topical collection which is edited by Aislinn Williams and Mark Niciu will be published soon in our journal. This will highlight different facets of stem cell research with emphasis upon translational neuroscience which has been hampered by lack of access to the affected tissue as well as insufficient animal models of complex neurological and psychiatric disorders, e.g. Alzheimer-type dementia and schizophrenia. These hurdles may be surmounted by recent advances in stem cell technologies, particularly the ability to reprogram differentiated cells like skin fibroblasts and lymphocytes from affected individuals. Pluripotent stem cells can differentiate along the neuroectodermal lineage into neurons and glia (astrocytes and oligodendrocytes), as well as other non-neuroectodermal cell types like microglia. Stem cell technologies can create enriched (> 90%) cell types, e.g., serotonergic, gamma-aminobutyric acid (GABA)-ergic and glutamatergic neurons, with brain-region specificity, e.g., neocortex, hippocampus, and cerebellum. Mixed organoid cultures which include functional cerebral-like vasculature will enable studies related to delivery of nutrients and elimination of waste products. This special issue will display stem cell-based technologies as critical tools in translational neuroscience.

## Connexins, innexins, and pannexins: from structure to physiology

The critical role of microscopy in elucidating ultrastructural features led to the identification and subsequent coining of the term “gap” junction in 1967 (Revel and Karnovsky 1967). Since the inception of this field, *Cell and Tissue Research* has published a wealth of studies examining the ultrastructure of these junctions in both vertebrate and invertebrate species. Connexin proteins are now recognized as the subunits that form gap junctions, facilitating direct intercellular communication essential for tissue development and homeostasis. In contrast, non-chordates, such as flatworms and *Drosophila*, utilize the innexin protein family for similar intercellular communication. More recently, pannexins have been identified in vertebrates, sharing homology with Innexins but primarily functioning as transmembrane channels that connect intracellular and extracellular environments.

The topical collection edited by Trond Aasen, James Smyth, and Silvia Penuela will highlight cutting-edge research and the ongoing evolution of the field. This includes significant progress in understanding channel structures through technological advancements like cryo-EM, exploring the non-canonical roles of these proteins in interactions with other cellular structures such as mitochondria, and their implications in an expanding array of physiological functions and diseases.

## Closing comments

We thank all past editors and authors who have guaranteed the continued success of our journal and we encourage our contemporary colleagues to submit their valuable research to CTR. In 2024, the journal expanded its focus to include the topic of cell and tissue response to infection; we look forward to publishing significant contributions from this field. We gaze into the future of Cell and Tissue Research with unwavering optimism and excitement. Innovative new technologies like the developments in high cellular resolution spatial proteomic/transcriptomic field, advances within genomics to decipher gene regulation networks and the application of artificial intelligence tools will unravel new dynamics in cell behavior. These types of studies will elucidate how distinct cell types interact and communicate within tissues, interact with the environment including microbes and interconnect within the whole organism. Such experiments will faciliate new discoveries and we hope that many of these findings will be communicated to the public via *Cell and Tissue Research* in the next 100 years!
